# Detection of SARS-CoV-2 Nucleocapsid, Spike, and Neutralizing Antibodies in Vaccinated Japanese

**DOI:** 10.3390/v14050965

**Published:** 2022-05-05

**Authors:** Rie Midorikawa, Moriyuki Nakama, Hiroshi Furukawa, Shomi Oka, Takashi Higuchi, Hideaki Nagai, Nobuhiro Nagai, Shigeto Tohma

**Affiliations:** 1 Department of Clinical Laboratory, National Hospital Organization Tokyo National Hospital, 3-1-1 Takeoka, Kiyose 204-8585, Japan; sugihara.rie.we@mail.hosp.go.jp (R.M.); nakama.moriyuki.vw@mail.hosp.go.jp (M.N.); nagai.nobuhiro.cy@mail.hosp.go.jp (N.N.); 2 Department of Clinical Laboratory, National Hospital Organization Shimofusa Psychiatric Medical Center, 578 Heta-cho, Midori-ku, Chiba 266-0007, Japan; 3 Department of Rheumatology, National Hospital Organization Tokyo National Hospital, 3-1-1 Takeoka, Kiyose 204-8585, Japan; oka-tkb@umin.org (S.O.); takashi.qef@ac.auone-net.jp (T.H.); touma.shigeto.jy@mail.hosp.go.jp (S.T.); 4 Clinical Research Center for Allergy and Rheumatology, National Hospital Organization Sagamihara National Hospital, 18-1 Sakuradai, Minami-ku, Sagamihara 252-0392, Japan; 5 Department of Nephrology, Ushiku Aiwa General Hospital, 896 Shishiko-cho, Ushiku 300-1296, Japan; 6 Department of Respiratory Medicine, National Hospital Organization Tokyo National Hospital, 3-1-1 Takeoka, Kiyose 204-8585, Japan; nagai.hideaki.gt@mail.hosp.go.jp

**Keywords:** vaccination, SARS-CoV-2 antibody, nucleocapsid, spike, neutralizing antibody

## Abstract

Serological detection of severe acute respiratory syndrome coronavirus 2 (SARS-CoV-2) nucleocapsid (N), spike (S), and neutralizing antibodies (Abs) is commonly undertaken to evaluate the efficacy of vaccination. However, the relative efficiency of different SARS-CoV-2 Ab detection systems has not been extensively investigated. Here, we evaluated serological test systems in vaccinated Japanese. SARS-CoV-2 N, S, and neutralizing Abs in sera of 375 healthy subjects a mean 253 days after vaccination were assessed. The sensitivity of Elecsys Anti-SARS-CoV-2 S (Roche S) and Anti-SARS-CoV-2 S IgG (Fujirebio S) was 100% and 98.9%, respectively, with a specificity of 100% for both. The sensitivity of Anti-SARS-CoV-2 neutralizing Ab (MBL Neu) was 2.7%, and the specificity was 100%. Fujirebio S correlated with Roche S (rho = 0.9182, *p* = 3.97 × 10^−152^). Fujirebio S (rho = 0.1295, *p* = 0.0121) and Roche S (rho = 0.1232, *p* = 0.0170) correlated weakly with MBL Neu. However, Roche S did correlate with MBL Neu in patients with COVID-19 (rho = 0.8299, *p* = 1.01 × 10^−12^) and in healthy subjects more recently after vaccination (mean of 90 days, rho = 0.5306, *p* = 0.0003). Thus, the Fujirebio S and Roche S results were very similar, but neither correlated with neutralizing antibody titers by MBL Neu at a later time after vaccination.

## 1. Introduction

Coronavirus disease 2019 (COVID-19) is caused by severe acute respiratory syndrome coronavirus 2 (SARS-CoV-2). An outbreak of COVID-19 was reported in December 2019 in Wuhan, China [1] and is ongoing worldwide. For the clinical diagnosis of COVID-19, the real-time reverse transcription polymerase chain reaction (RT-PCR) is performed to detect SARS-CoV-2 in samples from sputum, nasopharyngeal swabs or saliva [2]. Serological detection of SARS-CoV-2 antibodies (Abs) is also conducted to determine prior infection with the virus and to evaluate vaccine efficacy. The spike (S) and nucleocapsid (N) proteins of SARS-CoV-2 are target antigens for serological assays [3,4,5,6,7], because these proteins include immunogenic epitopes [8]. SARS-CoV-2 S Ab is commonly measured to evaluate vaccine efficacy, because most vaccines against the virus use S antigens for immunization. SARS-CoV-2 N Abs should be measured for the detection of prior infection in vaccinated subjects. On the other hand, neutralizing Abs are also analyzed to estimate the protective capacity of Abs against SARS-CoV-2. The plaque-reduction neutralization test (PRNT) using SARS-CoV-2 has been employed for this purpose, but it requires a biosafety level 3 facility. Thus, alternative methods have been developed using enzyme-linked immuno-sorbent assay (ELISA) to measure prevention of the receptor binding domain of S protein binding to human angiotensin converting enzyme 2. It was reported that ELISA-based neutralizing Ab assays correlated well with cell culture-based virus neutralization assays [9,10,11]. Additionally, this ELISA also correlated with SARS-CoV-2 S Ab detection assays [12]. However, correlations between SARS-CoV-2 Ab detection assays and the ELISA-based neutralizing Ab assay have not been extensively investigated in vaccinated Japanese. Hence, we compared serological testing for SARS-CoV-2 N, S, and neutralizing Abs in Japanese a relatively long time (about 8 months) after vaccination.

## 2. Materials and Methods

### 2.1. Patients and Sera

A total of 375 healthy subjects was recruited at Tokyo National Hospital, of whom 368 had been vaccinated twice against SARS-CoV-2 using BNT162b2 (Pfizer, New York City, NY, USA) between February 2021 and April 2021. Sera were collected from November 2021 to December 2021 before a third vaccination. None of the participants was diagnosed with COVID-19 before serum collection. Participants were 40.7 ± 12.0 years of age (mean ± SD), 26.9% were men [*n* = 101], and the time from the last vaccination was 252.6 ± 10.9 days. Sera from 42 of these participants were also collected in June 2021 (90.2 ± 15.4 days from the last vaccination). Additionally, 52 healthy unvaccinated subjects were recruited at Sagamihara National Hospital from July 2014 to October 2015 (pre-pandemic). COVID-19 patients were recruited at Tokyo National Hospital from April 2020 to February 2021 (*n* = 47, time from symptom onset: 16.7 ± 4.7 days). Sera from these participants were analyzed for SARS-CoV-2 Abs. This study was approved by The Research Ethics Committee of Tokyo National Hospital and Sagamihara National Hospital (Approval Code: 469, Approval Date: 25 March 2020), which waived the requirement for written informed consent from patients with COVID-19 under the regulations for emerging infectious diseases. Hence, oral informed consent was obtained from the patients with COVID-19, but written informed consent was obtained from the healthy subjects. This study was conducted in accordance with the principles expressed in the Declaration of Helsinki.

### 2.2. SARS-CoV-2 Ab Analyses

Antibodies of the IgG class specific for SARS-CoV-2 N and S were detected using chemiluminescent enzyme immunoassays (Anti-SARS-CoV-2 N IgG [Fujirebio N] and S IgG [Fujirebio S], Fujirebio, Hachioji, Japan), according to the manufacturer’s instructions. The cut-off value set by both manufacturers was 1.0 U/mL. IgM and IgG classes of SARS-CoV-2 S Abs were detected using the electrochemiluminescence immunoassay system Elecsys Anti-SARS-CoV-2 S [Roche S] (Roche Diagnostics, Mannheim, Germany), according to the manufacturer’s instructions. The cut-off value set by the manufacturer was 0.8 U/mL. SARS-CoV-2 neutralizing Abs were also detected using the blocking ELISA (SARS-CoV-2 neutralization Ab assay [MBL Neu], Medical & Biological Laboratories Co., Ltd., Tokyo, Japan), according to the manufacturer’s instructions (Supplementary Figure S1). The degree of inhibition by each sample was calculated as follows: inhibition (%) = (1 − optical density value of sample/optical density value of blank) × 100. Based on the 98th percentile among the 52 pre-pandemic healthy subjects from Sagamihara National Hospital, the cut-off value for positivity was set to 11.68% inhibition. SARS-CoV-2 Ab detection assays were not repeatedly performed. The results of SARS-CoV-2 S Ab assays using Roche S for COVID-19 patients were previously reported [13].

### 2.3. Statistical Analysis

Differences of Ab levels were analyzed by Mann–Whitney U test. The concordance between Ab detection assays was analyzed with overall, positive, or negative percent agreement values or Cohen’s kappa values. The area under the curve (AUC) values of the receiver operating characteristic (ROC) curves for Abs were calculated. Optimized cut-off levels, sensitivities, and specificities were calculated based on the highest Youden’s index from ROC curves. Correlations between Ab detection systems were evaluated with Spearman’s rho values.

## 3. Results

### 3.1. SARS-CoV-2 Abs in the Sera

SARS-CoV-2 N, S, and neutralizing Abs were investigated (Table 1). The difference between vaccinated and non-vaccinated participants using the Roche S test was highly significant (561.4 ± 431.5-vs.-0.4 ± 0.0, *p* = 5.80 × 10^−6^). The same was true for the Fujirebio S tested (15.6 ± 12.5-vs.-0.1 ± 0.0, *p* = 5.80 × 10^−6^). SARS-CoV-2 N Abs were not detected by Fujirebio N in any participants. SARS-CoV-2 neutralizing Abs detected by MBL Neu were not significantly different between vaccinated and non-vaccinated groups.

Clinical performance of the SARS-CoV-2 Ab assays is described in Table 2. The sensitivity of Roche S and Fujirebio S was 100% (95% confidence intervals [CI] 99.0–100.0%) and 98.9% (95%CI 97.2–99.7%), respectively. The specificity of Roche S and Fujirebio S was 100% (95%CI 59.0–100.0%) for both. The sensitivity of MBL Neu was 2.7% (95%CI 1.3–4.9%) and the specificity was 100% (95%CI 59.0–100.0%). The concordance between SARS-CoV-2 Ab assays is shown in Table 3. The overall percent agreement between Roche S and Fujirebio S was 98.9% (95%CI 97.3–99.7%). The positive percent agreement between the two was 98.9% (95%CI 97.2–99.7%) and the negative percent agreement value was 100.0% (95%CI 59.0–100.0%). Cohen’s kappa value between them was 0.773 (95%CI 0.557–0.988). in contrast, the overall agreement between Fujirebio S and MBL Neu was only 5.6% (95%CI 3.5–8.4%), the positive percent agreement was 2.7% (95%CI 1.3–5.0%) and the negative percent agreement was 100.0% (95%CI 71.5–100.0%). Cohen’s kappa value between Fujirebio S and MBL Neu was 0.002 (95%CI 0.000–0.003). Similarly, the overall agreement between Roche S and MBL Neu was 4.5% (95%CI 2.7–7.2%), the positive percent agreement was 2.7% (95%CI 1.3–4.9%) and the negative percent agreement was 100.0% (95%CI 59.0–100.0%). Cohen’s kappa value between Roche S and MBL Neu was 0.001 (95%CI 0.000–0.002).

### 3.2. ROC Analyses

**Figure 1:**
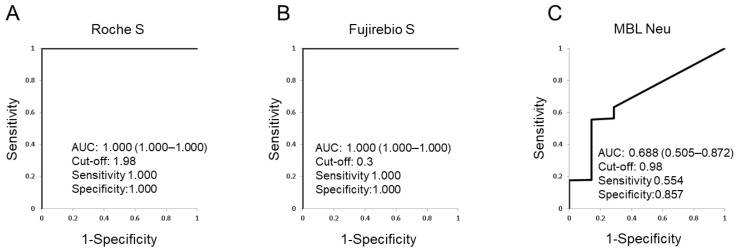
Receiver operating characteristic (ROC) curve analyses of SARS-CoV-2 Abs. ROC curves for Roche S (**A**), Fujirebio S (**B**), and MBL Neu (**C**). The area under the curve (AUC) values of the ROC curves with 95% confidence intervals and the optimized cut-off levels with specificities and sensitivities are depicted. ROC: receiver operating characteristic, AUC: area under the curve. SARS-CoV-2: severe acute respiratory syndrome coronavirus 2, Roche S: Roche Elecsys anti-SARS-CoV-2 S, Fujirebio S: Fujirebio SARS-CoV-2 S IgG, MBL Neu: MBL Anti-SARS-CoV-2 neutralization Ab.

ROC analyses were performed for the SARS-CoV-2 Ab assays and AUC values were calculated (Figure 1). The AUC value for Roche S and Fujirebio S was 1.000 (95%CI 1.000–1.000) for both, whereas the AUC value for MBL Neu was 0.688 (95%CI 0.505–0.872). The optimized cut-off value of Roche S was 1.98, which is higher than set by manufacturer, whereas for Fujirebio S it was 0.3, lower than set by manufacturer. The optimized cut-off value of MBL Neu was 0.98%, lower than set based on the 98th percentile among the 52 pre-pandemic healthy subjects.

### 3.3. Correlations between SARS-CoV-2 Ab Assays

**Figure 2:**
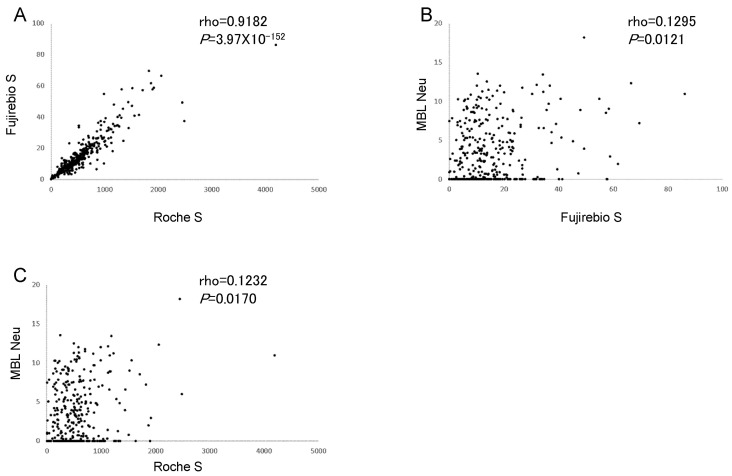
Correlations of SARS-CoV-2 Ab measurements. Spearman correlations between Fujirebio S and Roche S (**A**), MBL Neu and Fujirebio S (**B**), and MBL Neu and Roche S (**C**). Roche S: Roche Elecsys anti-SARS-CoV-2 S, Fujirebio S: Fujirebio SARS-CoV-2 S IgG, MBL Neu: MBL SARS-CoV-2 neutralizing Ab test.

Correlations between results from the SARS-CoV-2 S Ab assays are shown in Figure 2. The Fujirebio S assay strongly correlated with Roche S (rho = 0.9182, *p* = 3.97 × 10^−152^), and both weakly correlated with MBL Neu (rho = 0.1295, *p* = 0.0121 and rho = 0.1232, respectively, *p* = 0.0170). These data confirm the strong correlation between Roche S and Fujirebio S assays.

Correlations between results from Roche S and MBL Neu in other populations were additionally analyzed (Supplementary Table S1 and Figure S2). Roche S strongly correlated with MBL Neu in patients with COVID-19 (rho = 0.8299, *p* = 1.01 × 10^−12^). Additionally, Roche S moderately correlated with MBL Neu in healthy vaccines when the time between the last vaccination and serum collection was shorter (mean 90 days rather than 253 days; rho = 0.5306, *p* = 0.0003). These data document a stronger correlation between Roche S and MBL Neu in patients with COVID-19 or healthy subjects vaccinated more recently, which wanes with increasing time after vaccination.

## 4. Discussion

Here, we evaluated the clinical performance of SARS-CoV-2 Ab assays in vaccinated Japanese a relatively long time after vaccination (8 months). The sensitivity and specificity of Roche S and Fujirebio S were both ≥98% (Table 2), although these values were lower for the MBL Neu assay for neutralizing Abs. Additionally, SARS-CoV-2 N Abs were not detected by Fujirebio N in any of the participants. Because vaccination against SARS-CoV-2 generates SARS-CoV-2 S Abs, SARS-CoV-2 N Abs should be used for the detection of prior infection in vaccinated individuals. In this study, it was also shown that Roche S test results strongly correlated with those of Fujirebio S (Table 3 and Figure 2A), suggesting similar clinical performance of the two systems. Although the clinical performance of MBL Neu was poorer in vaccinated Japanese at a longer time after vaccination (Figure 1C and Figure 2B,C), it was better more recently thereafter, as well as in COVID-19 patients (Supplementary Table S1 and Figure S1). Additionally, MBL Neu and PRNT were shown to correlate well when tested side-by-side in patients with actual COVID-19 [14]. These data suggested that levels of neutralizing Abs are lower a longer time after vaccination, compared with SARS-CoV-2 S Abs. It was reported that the production of neutralizing Abs was maintained for several months after infection with SARS-CoV-2 [15,16]. However, the levels of neutralizing Abs were decreased after 9 months in vaccinated individuals [17]. The results obtained in the present study are consistent with these reports. Although SARS-CoV-2 S Abs were detected in sera from subjects a longer time after vaccination, the levels of neutralizing Abs decreased; the weaker correlation of MBL Neu with Fujirebio S or Roche S was due to the decreased neutralizing Ab titers a longer time after vaccination.

Correlations between the results of several assays for SARS-CoV-2 Abs were reported to be strong in patients with COVID-19 [12]. That study found that SARS-CoV-2 S Ab detection assays including Roche S and an ELISA-based neutralizing Ab assay (cPass, GenScript) were well-correlated. In the present study, Fujirebio S correlated with Roche S, but did not with MBL Neu longer after vaccination. These data suggest that Fujirebio S would also correlate with those assays reported in the previous study [12]. The sensitivity of Fujirebio S was slightly lower than Roche S (Table 2), suggesting that the optimized cut-off level of Fujirebio S was lower than the manufacturer’s recommended cut-off levels (Figure 1B). This in turn suggests that the cut-off level of Fujirebio S was defined to decrease false positive rates. Although IgM and IgG levels are measured by Roche S, only IgG levels are detected by Fujirebio S. The results were strongly correlated between the two test systems, because IgM levels would be low such a long time after the last vaccination.

The present report on the serological testing for SARS-CoV-2 N, S, and neutralizing Abs in vaccinated Japanese a longer time after vaccination has some limitations. The titers of SARS-CoV-2 S Abs in the participants tested here were relatively low due to the long time from the last vaccination (Table 1). Serum samples with higher titers of SARS-CoV-2 S Abs, i.e., the sera from more recently vaccinated participants, should be analyzed for comparing the four SARS-CoV-2 Ab assays. In the present study, four SARS-CoV-2 Ab detection assays were evaluated, but other new or commonly used assays could be also compared using the same sample sets. Four SARS-CoV-2 Ab assays should be compared with serum samples from COVID-19 patients. The comparison of vaccinated and non-vaccinated subjects was skewed (Table 2) because non-vaccinated subjects were rare. In the present study, T cell function stimulated by SARS-CoV-2 in vaccinated participants was not evaluated, although the role of T cells cannot be ignored in immunological systems [18]. This study evaluated the clinical performance of SARS-CoV-2 Ab assays in vaccinated Japanese a relatively long time after vaccination. Roche S correlated with Fujirebio S in vaccinated Japanese, though MBL Neu did not. These results extend the application of serological COVID-19 tests.

## Figures and Tables

**Table 1:** SARS-CoV-2 Ab titers.

	Vaccinated Subjects	Non-Vaccinated Subjects	*p*
Number	368	7	
Roche S, U/mL (SD)	561.4 (431.5)	0.4 (0.0)	5.80 × 10^−6^
Fujirebio N, AU/mL (SD)	0.0 (0.0)	0.0 (0.0)	0.9748
Fujirebio S, AU/mL (SD)	15.6 (12.5)	0.1 (0.0)	5.80 × 10^−6^
MBL Neu, inhibition rate (%) (SD)	3.3 (3.8)	1.2 (2.8)	0.0792

Number or Average values of each group are shown. Standard deviations are shown in parentheses. Differences were tested by Mann–Whitney U testing. SARS-CoV-2: severe acute respiratory syndrome coronavirus 2, Roche S: Roche Elecsys Anti-SARS-CoV-2 S, Fujirebio N: Fujirebio SARS-CoV-2 N IgG, Fujirebio S: Fujirebio SARS-CoV-2 S IgG, MBL Neu: MBL SARS-CoV-2 neutralizing Ab, SD: standard deviation.

**Table 2:** Clinical performance of SARS-CoV-2 Ab assays.

		Vaccinated Subjects	Non-Vaccinated Subjects	Sensitivity (95%CI)	Specificity (95%CI)
Roche S	positive	368	0	100.0 (99.0–100.0)	100.0 (59.0–100.0)
	negative	0	7		
Fujirebio S	positive	364	0	98.9 (97.2–99.7)	100.0 (59.0–100.0)
	negative	4	7		
MBL Neu	positive	10	0	2.7 (1.3–4.9)	100.0 (59.0–100.0)
	negative	358	7		

Number of participants in each group is shown. SARS-CoV-2: severe acute respiratory syndrome coronavirus 2, Roche S: Roche Elecsys Anti-SARS-CoV-2 S, Fujirebio S: Fuji-rebio SARS-CoV-2 S IgG, MBL Neu: MBL SARS-CoV-2 neutralizing Ab, CI: confidence intervals.

**Table 3:** Concordance between SARS-CoV-2 Ab detection assays.

	**Fujirebio S Compared with Roche S**		**MBL Neu Compared with Fujirebio S**		**MBL Neu Compared with Roche S**	
OPA, *n*, %, (95%CI)	371/375	98.9 (97.3–99.7)	21/375	5.6 (3.5–8.4)	17/375	4.5 (2.7–7.2)
PPA, *n*, %, (95%CI)	364/368	98.9 (97.2–99.7)	10/364	2.7 (1.3–5.0)	10/368	2.7 (1.3–4.9)
NPA, *n*, %, (95%CI)	7/7	100.0 (59.0–100.0)	11/11	100.0 (71.5–100.0)	7/7	100.0 (59.0–100.0)
Cohen’s kappa, (95%CI)		0.773 (0.557–0.988)		0.002 (0.000–0.003)		0.001 (0.000–0.002)

Number of participants in each group is shown and 95%CI values are shown in pa-rentheses. SARS-CoV-2: severe acute respiratory syndrome coronavirus 2, Roche S: Roche Elecsys Anti-SARS-CoV-2 S, Fujirebio S: Fujirebio SARS-CoV-2 S IgG, MBL Neu: MBL SARS-CoV-2 neutralizing Ab, OPA: overall percent agreement, PPA: positive percent agreement, NPP: negative percent agreement, CI: confidence intervals.

## Data Availability

All data are presented in the paper.

## Supplementary Materials

The following supporting information can be downloaded at: https://www.mdpi.com/article/10.3390/v14050965/s1, Figure S1: The correlation of anti-SARS-CoV-2 Abs; Figure S2: The correlation of anti-SARS-CoV-2 Abs; Table S1: SARS-CoV-2 Abs of COVID-19 patients and healthy subjects with a shorter interval between last vaccination and serum collection.
