# Supplementary material for: Detection of SARS-CoV-2 Nucleocapsid, Spike, and Neutralizing Antibodies in Vaccinated Japanese
Source: Viruses. 2022 May 5;14(5):965. doi: 10.3390/v14050965 (PMC9144302; doi:10.3390/v14050965)
Supplement: Supplementary file 1 [file viruses-14-00965-s001.zip › supplementary-viruses-1651220.pdf]

Supplementary Table S1. SARS-CoV-2 Abs of COVID-19 patients and healthy subjects with a shorter interval between last vaccination and serum collection.

	COVID-19 patients	Healthy subjects 90 days after vaccination
Number	46	42
Roche S, U/ml (SD)	129.9 (287.7)	1269.1 (1065.9)
Roche S positive, n (%)	37 (80.4)	42 (100.0)
MBL Neu, inhibition rate (%) (SD)	22.2 (21.1)	8.4 (8.2)
MBL Neu positive, n (%)	28 (60.9)	13 (31.0)

Number or average values of each group are shown. Standard deviations or percentages are shown in parentheses. COVID-19: coronavirus disease 2019, SARS-CoV-2: severe acute respiratory syndrome coronavirus 2, Roche S: Roche Elecsys anti-SARS-CoV-2 S, MBL Neu: MBL Anti-SARS-CoV-2 neutralization Ab.

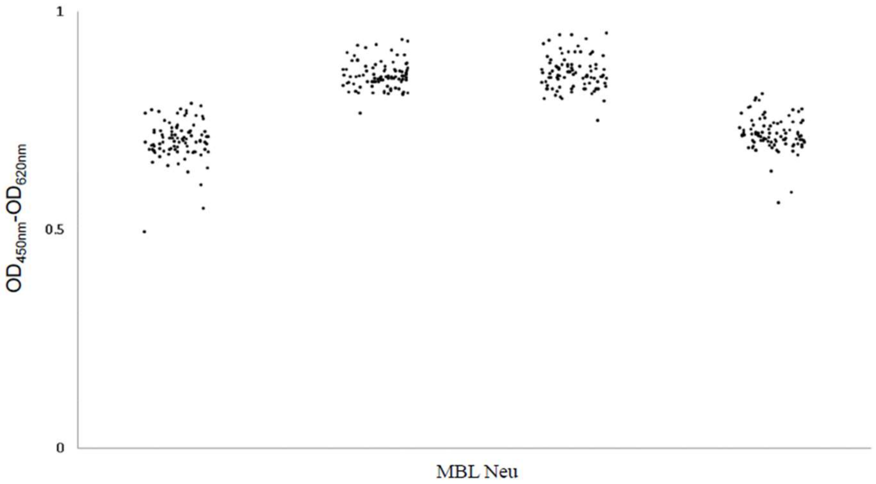

Supplementary Figure S1. The OD values in the detection of anti-SARS-CoV-2 neutralizing Abs. OD values of four experiments in the detection of anti-SARS-CoV-2 neutralizing Abs using MBL Neu. SARS-CoV-2: severe acute respiratory syndrome coronavirus 2, MBL Neu: MBL Anti-SARS-CoV-2 neutralization Ab, OD: optical density.

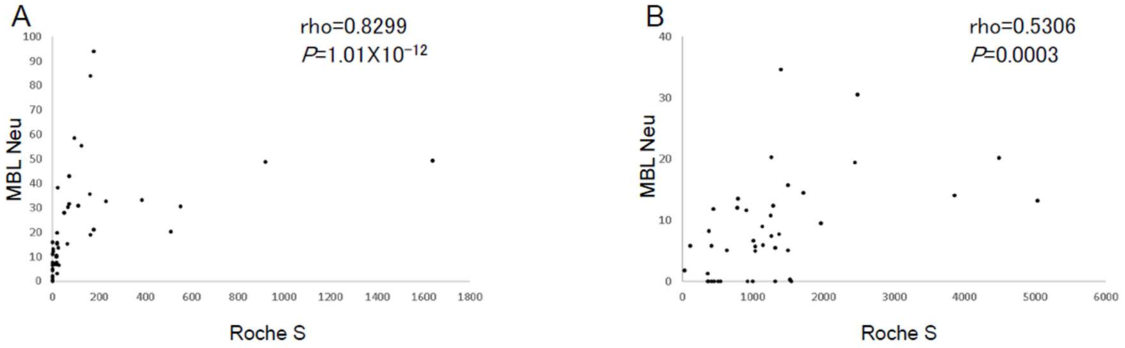

Supplementary Figure S2. The correlation of anti-SARS-CoV-2 Abs. Spearman correlation between Roche S and MBL Neu for COVID-19 patients (A) and healthy subjects with shorter intervals between last vaccination and serum collection (B). SARS-CoV-2: severe acute respiratory syndrome coronavirus 2, Roche S: Roche Elecsys anti-SARS-CoV-2 S, MBL Neu: MBL Anti-SARS-CoV-2 neutralization Ab.
